# Studies on bioactivities of Manuka and regional varieties of honey for their potential use as natural antibiotic agents for infection control related to wound healing and in pharmaceutical formulations

**DOI:** 10.3934/microbiol.2024015

**Published:** 2024-05-06

**Authors:** Divakar Dahiya, Caoimhin Mackin, Poonam Singh Nigam

**Affiliations:** 1a Wexham Park Hospital, Wexham Street, Slough SL2 4HL, England, UK; 1b current address: Haematology and Blood Transfusion, Basingstoke and North Hampshire Hospital, Basingstoke RG24 9NA, UK; 2 Biomedical Sciences Research Institute, Ulster University, Coleraine BT52 1SA, UK

**Keywords:** infection, honey, Manuka, bioactivity, antibiotic, antibacterial, antifungal, wound healing, pharmaceutical

## Abstract

Presently, most of the reported infections are of a bacterial origin; however, this leads to a limit within the literature and research around infections caused by fungal pathogens, which are now developing resistance to antibiotic medicines. Of the natural antimicrobial agents, honey has been observed with demonstrable and highly exploitable antimicrobial and infection control related to wound healing properties; therefore, it has been incorporated into many standard pharmaceutical formulations. Generally, these products utilize a pure sample of honey as a bioactive ingredient in a product which has been purposely designed for the convenience of application. This article aims to review information available from published reports on various bioactivities of a variety of medical-grade honey products, including manuka and other conventional non-manuka types sourced from different floral types and geographical regions. Additionally, this review highlights the antibiotic activities of various types of honey products tested against pathogenic strains of bacteria, yeast and fungi, and their applications in the formulation of healthcare products.

## 1. Introduction

The World Health Organization published a global report in 2022, which reviewed and emphasized the importance of a specific clinical procedure known as Infection Prevention and Control (IPC). This procedure has been defined in the previously mentioned report as a clinical and public health specialty, based on an evidence-centered and practical approach, which focused on the prevention of harm done due to avoidable infections in patients, healthcare workers, and visitors to such facilities. In most cases, it was found that infections developed from antibiotic-resistant pathogens during the provision of clinical and healthcare services [1-i]. According to the report, a brief was prepared by the WHO and the Organization for Economic Co-operation and Development (OECD) for the German Presidency in 2022 to develop an analysis of IPC programs and their activities [1-ii]. The brief was required in recognition of a need to strengthen the IPC capacity in healthcare facilities, which could help to set a strategy to reduce the spread of antimicrobial resistance and infections associated with healthcare.

In a multi-center study, the epidemiology of healthcare-associated infections (HCAIs) and the mechanisms of antimicrobial resistance of responsible pathogens were investigated. Persons infected with an antibiotic-resistant pathogen could be influenced by a two- to three-fold increased mortality rate, compared to those people infected with a non-resistant pathogen [2]. A recently published report was based on an investigation of the incidence and impact of infections on patients that were primarily admitted with diagnoses of sepsis and non-sepsis. It was noticed that, specifically, one in four hospital-treated sepsis patients happened to have an HCAI, and one in two adult intensive-care-unit sepsis patients also had an HCAI [3].

In a report published by the European Centre for Disease Prevention and Control, which assessed the health burden of infections with antibiotic-resistant bacteria in the EU/EEA, 2016–2020, it was found that 70.9% of infections across the European Union between the years 2016 and 2019 that were of antibiotic-resistant bacteria in origin were HCAIs [4]. Four bacterial species, all of them being antibiotic resistant, were named in the 2022 WHO report as being the most detrimental to healthcare workers and patients across the European Union, specifically resistant to third-generation cephalosporin *Escherichia coli* and *Klebsiella pneumoniae*, Methicillin-Resistant *Staphylococcus aureus*, and Carbapenem-Resistant *Pseudomonas aeruginosa* [1-i,ii]. Of a particular note, these named pathogens, as well as the other antibiotic-resistant species of bacteria, impart an extra source of health hazard and complication within the practices of infection control, and their influence is felt on a global scale wherein the health of a person or a patient is a top priority. To tackle such a crisis, current programs of infection control must be reviewed and updated accordingly, and newer careful and responsible management programs need to be set to review the practice of prescribing chemical antibiotics to control the further development of antibiotic resistance in pathogens [5].

This article is based on a review of published reports on bioactivities possessed by honey, which is a natural nutraceutical substance. This review aims to present a concise description of various therapeutic activities, including antioxidant, antibacterial, and antifungal, in several types of honey sourced from different floral varieties and geographical regions, and its possible application as an active antibiotic ingredient in healthcare products.

## 2. Control of infections

Specifically, the management of antibiotic-resistant infections and the methods which should be conducted for the treatment are a priority that must be addressed. A recent review published by Seidelman et al. reported that 0.5–3% of all patients who undergo or are undergoing a surgical procedure will develop a surgical wound infection or an infection adjacent to the wound itself. That might/will reduce total wound healing and will result in an increased admittance time within the hospital as compared to those patients without surgical wound infections. Such occurrences of after-infections add costs, resources, and staffing burdens to the hospital that it must make up for, while simultaneously treating surgery patients and nosocomial infections [6].

Similarly, another review conducted by Sen illustrated the exact financial burden that human wound care exerted on healthcare systems. Up to 2.5% of the total US population's quality of life is negatively impacted by mandatory wound healing and its management, chronic wound management, and chronic non-healing wound management. Of the wound types, their subsequent care and wound infection management, specifically surgical wound infection management, was the most expensive [7]. The direct consequences of the COVID-19 pandemic on such treatments regiments as wound care, highlighting that widespread inappropriate wound healing was a result of the disruption of standard procedure wound care continuity, with which the healthcare system is now burdened to work back within a post-pandemic world and system. During the pandemic, routine outpatient appointments, such as wound care-specific appointments, were considered either non-essential or had limited access due to obvious reasons [7],[8].

Wound healing, which is the management and treatment of chronic wounds, costs the National Health Service (NHS) around £8.3 billion [9]; these services predominantly focus on the treatment and eradication of any potentially stagnating bacteria, especially known biofilm-forming species such as *Pseudomonas aeruginosa*, that may be present within a healing wound, especially within the inflammatory stages of healing. However, many of these procedures and treatment regimens fail to address potential fungal pathogens that may be present within a wound environment. A study conducted by Dowd et al. evaluated 915 chronic wound specimens for the potential incidence, abundance, and species diversity of fungi and concluded that 208 (23%) of the pathology specimens were positive for the presence of fungal species – namely *Candida* sp., *Curvularia sp., Malessezia sp., Aureobasidium sp., Cladosporium sp., Ulocladium sp., Engodontium sp*., and *Trichtophyton* [10].

In an investigation of the spectrum of potential infectious microflora, Bansal et al evaluated 103 patients with diabetic foot ulcers, which is a form of chronic wound. During the identification of microbiota isolated from the specimens, the prevalence of fungal isolates up to 9% was found, of which *Candida albicans* and *Aspergillus flavus* were the most common in 9% of the fungal-infected samples [11]. The frequency of both fungal and bacterial pathogens within wound environments that possess inappropriate healing are not exclusive consequences. The implications of fungal pathogens within wounds, whether they are chronic or not, can no longer be underestimated. Two recent studies reported that fungal colonization of a wound assisted opportunistic bacteria within a non-healing wound. Infections evade the host's immune system detection, as well as reduce the efficacy of antibiotics, facilitating such wounds to become reservoirs to potentially multi-drug resistant pathogens [12],[13].

In a recently published report by Vitillo et al, with continuously immerging resistance in pathogenic strains the antifungal drug resistance has been reported as an emergent health threat for antibiotics and for new antimycotic substances [14]. Hence, the research and development of novel wound care agents must be undertaken with urgency to reduce this rising threat to public health and the healthcare system. The potential of some natural materials has been tested, including sampling materials of aquatic and land origins [15],[16], insect origins [17],[18], and plant origins [19],[20]. The promising material of this plethora of natural products was found to be honey [21].

## 3. A natural antibiotic agent honey

Honey is a natural material of a thick consistency, is golden brownish in color, and has a very sweet taste. It is produced after the digestion and processing of flower nectar within the upper digestive tract of honeybees of the genus *Apis*. Beyond the high concentration of hexose sugars, the exact composition of a sample of honey depends on the following three main criteria:

i. Geographical location;

ii. Floral species whose nectar was harvested by bees; and

iii. The species of honeybee that harvested the nectar.

However, few general qualities remain the same between all varieties of honey, whether Manuka or non-Manuka; that is an approximately 80% hexose sugar composition and 20% water content, with varying compounds such as minerals, vitamins, enzymes, amino acids, peptides, flavonoids, phenolic acids, and other volatile phytochemicals [22].

**Figure 1.**
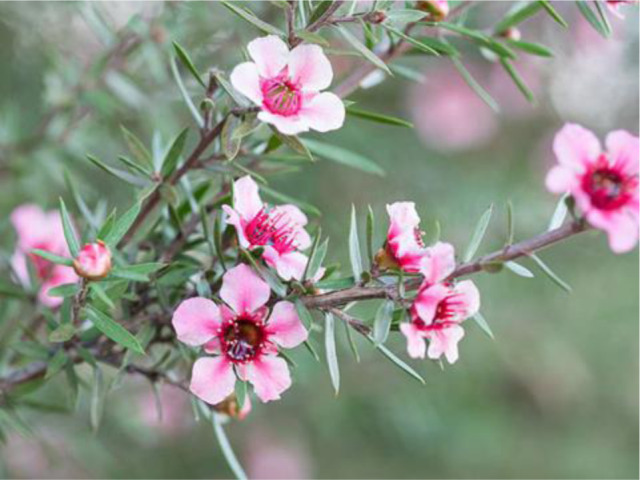
*Leptospermum scoparium*, commonly referred to as Manuka Myrtle, Manuka, New Zealand Tea Tree, or just Tea Tree. Source of image: https://www.gardenia.net/plant/leptospermum-scoparium [accessed on 14/07/2023].

Of all the diverse varieties of honey examined, one type that has been shown to possess the most demonstrable exploitable properties is Manuka honey. Hegazi et al mentioned it as a super-food and the authors presented a comprehensive review of its analysis and the authenticity approaches for manuka honey [23]. Manuka honey is a specific variety of honey produced by the *Apis mellifera* species of honeybee. Manuka is classified as mono-floral (Figure 1) in origin due to the processing of nectar from a single species of a New Zealand Manuka tree, *Leptospermum scorparium*.

In recent decades, the study of manuka honey has emerged in several clinical investigations. Reports have classified manuka as medical-grade honey used alone or in combination with other disinfectants [24]. A variety of honey defined as medical-grade must be organic and should have been collected in a region free of contaminants. The collected product must be processed under standardized conditions to comply with legal and safe physicochemical properties, which are required for its use in antimicrobial activities [25]. In view of such guidelines for a medical grade product, distinct antimicrobial properties have been observed in manuka honey against biofilm-forming, non-antibiotic-resistant, bacteria species, as well as some antibiotic-resistant bacteria such as methicillin-resistant *Staphylococcus aureus* (MRSA) [25],[26]. A recent study was conducted to compare the antibacterial activity of Algerian Honey and Manuka Honey toward pathogenic bacteria from burn wound infections within the measure of wound care and wound treatment programs [27].

Manuka honey is categorized for its strength in the expression of bioactivities by its Unique Manuka Factor (UMF), which is a classification system that reflects the equivalent concentration of phenol (%, w/v) required to produce the same antibacterial activity as honey. However, the UMF rating may not be a reliable indicator of the actual antibacterial effect produced by honey, as it has been suggested that the bacteriostatic properties in Manuka honey are associated with the presence of a unique constituent known as methylglyoxal (MGO). Therefore, the UMF grading system reflects the concentration of MGO present in a particular type of commercially sold honey [28]. Manuka honey can demonstrate such a propensity for its bioactivity due to its MGO content. This compound is detected in higher concentrations within the Manuka variety compared to other non-manuka types of honey [28],[29], and is known for its characteristic antibacterial activity [30]. Manuka honey's UMF rating measures both the quality and purity of Manuka honey based on the levels of three unique compounds found in manuka: MGO, dihydroxyacetone (DHA), and leptosperin. UMF strongly correlates with MGO equivalence. A correlation between the two rating criteria is presented in Table 1.

**Table 1.** Rating of Manuka Honey in comparative values of two types of Grading system*.

Activities	Unique Manuka Factor (UMF)	Methylglyoxal Content (MGO)
A natural product for General usage	2.8+	30+
General Health Maintenance	6.0+	100+
Healthcare for Oral and Wound	10+	250+
Digestive Health Maintenance	15+	550+
Higher level of Healthcare for Oral and Wound	20+	850+
Super Manuka Grade	25+	1200+
Ultimate Potency	28+	1500+

*Source of information: www.australiasmanuka.com.au/medical-grade-honey-uses/

Manuka honey's UMF grade corresponds to its non-peroxide antimicrobial property. If a type of Manuka honey is labelled with a UMF rating of 10, it contains a non-peroxide antimicrobial property equivalent to a 10% phenol control solution. The honey with a higher UMF rating represents higher antibacterial properties [31],[32]. Honey is found to be effective against a wide range of pathogens, and the different UMF/MGO values of Manuka honey have differing effects on Gram-negative bacteria compared to Gram-positive bacteria. Based on the bioactive and antimicrobial properties, the proficiency of Manuka honey is so well known and clinically evaluated to such a degree; this material is used to act as a common comparator when screening and evaluating newer described varieties of honey from other non-Manuka uniflorous medicinal plants from a geographical diversity [31]–[34].

Despite the proficiency to produce antibacterial effects, there is limited literature on the application of honey specifically on fungal and yeast pathogenic species. This is a valid reason to prompt more detailed clinical research into honey's effect on wounds infected generally with non-bacterial pathogens, and the potential applications of honey on microbial infections caused by yeast, bacteria, and fungal pathogens.

## 4. Antibacterial and other Bioactivities in honey sourced from diverse geographical regions

Several studies have reported a variety of pharmaceutical, biochemical, and potential therapeutic properties in honey. Information collected from published research has demonstrated the antibacterial and antimycotic potential in the field of developing novel treatments for microbial infections. Table 2 summarises relevant studies on the antimicrobial activities of honey against common pathogens.

**Table 2.** Studies on Therapeutic properties of manuka and non-manuka honey.

Honey Types for Evaluation of Bioactive Properties	Reference
*Varieties of honey*:
Topical honey and povidone iodine-based dressings were reported effective for wound healing	[21]
*Manuka and non-manuka*: Antimicrobial potential of antibiotics were found effective in synergistic effects with honey against biofilms	[22]
*Manuka*: Manuka addressed as super-food honey possessed antibacterial activities	[23]
*Manuka*: Microbial wound surface infection healing was studied with honey alone or in combination with other disinfectants	[24]
*Four well-characterised New Zealand honey*: inhibition of infection and elimination of biofilms produced by *Pseudomonas aeruginosa* were detected	[25]
*Manuka*: Results showed inhibition of bacterial cell division in methicillin-resistant *Staphylococcus aureus*	[26]
of *Algerian honeys and manuka honey*
Antibacterial activity was reported toward pathogenic bacteria isolated from burn wound infections	[27]
*Varieties of honey*: Antibacterial potency was evaluated corresponding to its constituents	[28]
*Several varieties of* honey: Report suggested honey as an alternative remedy in the fight against antibiotic-resistant bacteria	[29]
*Manuka*: Antibacterial activity was evaluated in reference to the components in honey	[30]
*Four types of monofloral honey (Manuka, Brassica rapeseed, Acacia, and Linden)*:
Results demonstrated that antimicrobial and antioxidant potential varied with the diversity of monofloral honey	[31]
*Eight kinds of medicinal plant-derived uniflorous honey, native to China*:
Chemical profile of uniflorous honey from a medicinal plant, *Scrophularia ningpoensis* Hemsl showed potential for their antibacterial activity	[32]
*Regional varieties of honey: sourced from the Lemnos island of Greece and manuka from New Zealand*: Samples of honey with geographical diversity were compared for their antimicrobial actions against clinically important bacteria	[33]
*Nine kinds of uni-floral honey sourced from China and Manuka of UMF 12+ and 20+*:
Antimicrobial activity was measured with respect to the analysis conducted for the chemical constituents of honey varieties used in study	[34]
*Manuka-type honeys*: These types showed a potential for the management of wound healing as an anti-biofilm agent for eradicating biofilms produced by *Staphylococcus aureus* strains possessing different biofilm-forming abilities	[35]
*Manuka*: The cytotoxicity was induce against MCF7 breast cancer cells, which was correlated to the total phenol content and antioxidant activity analysed in the samples of manuka honey.	[36]
*Manuka*: Estrogenic and antiproliferative activity in honey tested *in* vitro were found cytotoxic to MCF-7 breast cancer cells	[37]
*Manuka*: Antioxidant, anticancer, antibacterial, antibiofilm properties were demonstrated in honey with respect to its chemical analysis	[38]
*Manuka*: Chemical constituents of natural unfractionated form of manuka honey were analysed for total phenols, in relation to its antioxidant capacity and antibacterial activity	[39]
*Manuka*: Extracts prepared from honey were analysed for total phenols, in relation to antioxidant capacity and antibacterial activity in extracts prepared from manuka honey	[40]
*Manuka*: Potent antioxidant iron (III) reducing capacity in pure unfractionated and fractionated forms of manuka was evaluated in comparison with ABTS and other methods	[41]
*Methylglyoxal susceptibility*: The effect of MGO content was tested on multidrug-resistant *Pseudomonas aeruginosa*	[42]
*Manuka*: A 5.8-kDa component of honey stimulated immune cells via TLR4 pathway	[43]
*Manuka, Kanuka, Acacia, Jungle, Honeydew Honey*: Components tested and evaluated for the improvement of wound healing in both chronic and acute conditions	[44]
*Commercial manuka and Leptospermum honeys from Australia and New Zealand*: The correlation of antibacterial activity was studied with its methylglyoxal content and other physicochemical characteristics	[45]
*Jarrah honey, Medihoney®, Comvita® Wound Care 18+, and an artificial honey*:
Broad-spectrum antimycotic and antifungal capacity were reported against clinically significant strains of *Candida* species including clinical isolates of *C. albicans, C. glabrata* and *C. dubliniensis*	[46]
*New honey varietals unifloral (Plum, Rapeseed, Lime, Phacelia, Honeydew, Sunflower, Willow, and multifloral (-P,-AP,-Sa -Br):*
Antimicrobial activity of new honey varietals was reported against
Bacteria: *Escherichia coli, Bacillus circulans, Staphylococcus aureus, Pseudomonas aeruginosa*,
Yeasts: *Saccharomyces cerevisiae* and *Candida albicans*
Fungi: *Aspergillus niger*	[47]
*Local Commercial honey from different parts of Nigeria*:
Antifungal capacity was reported *in-vitro* against *Aspergillus niger, A. flavus, Penicillium chrysogenum, Microsporum gypseum, Candida albicans*, and *Saccharomyces sp.*	[48]
*Commercial honey-based gel containing 40% honey*:
*In vitro* antibiotic efficacy was evaluated against canine clinical isolates of *Staphylococcus pseudintermedius* and *Malassezia pachydermatis*	[49]
*Medical grade honey L-Mesitran® Soft*: Antifungal activity of honey formulation was found effective against a pathogenic yeast *Candida auris*	[50]
*Honey from stingless bees Melipona beechii*: Antifungal activity was found effective against *Candida albicans*	[51]
*Portuguese honeys from Heather (Erica cinereal):*
Results concluded these honey being a suitable antimicrobial agents against *Candida* species	[52]
*Three Local Malaysian Honeys (Tualang, Acacia, and Kelutut)*:
Antifungal effect was demonstrated by these conventional varieties of honey on selected pathogenic fungi of otomycosis including *Aspergillus niger* and *Candida albicans*	[53]
*Romanian Honey and Propolis*:
Antifungal activity was reported in honey samples against six fungal strains: *Aspergillus niger, A. flavus, Candida albicans, Penicillium chrysogenum, Rhizopus stolonifer, Fusarium oxysporum*	[54]
*Honey products in Benin*: Antioxidant and antifungal activities were tested against four reference fungi: *Aspergillus parasiticus* CMBB 20, *Aspergillus ochraceus* CMBB 91, *Aspergillus fumigatus* CMBB 89, and *Aspergillus clavatus* NCPT 97	[55]
*Malaysian Stingless Bee Kelulut Honey*:
Regional variety of honey was evaluated for its potential use in wound dressings to improve healing of wounds in diabetic foot ulcers, study conducted on thirty adult diabetic patients suffering from chronic diabetic foot ulcers	[56]
*Latvian Honey*: antimicrobial and antibiofilm properties were shown by Latvian honey against causative agents of wound infections, including *Escherichia coli, Pseudomonas aeruginosa, Staphylococcus aureus*, clinical isolates Extended-Spectrum Beta-Lactamases producer *Escherichia coli*, Methicillin-resistant *Staphylococcus aureus* and *Candida albicans*.	[57]
*Talh Honey*: Formulation of Talh honey with whey protein and collagen was studied on acute excisional skin wound healing in Wistar male rat	[58]
*Pine honey produced across regions in Greece*:
Biological properties were tested against five nosocomial and foodborne pathogens, including *Klebsiella pneuomoniae, Salmonella ser. typhimurium, Staphylococcus aureus,Aspergillus baumannii, Pseudomonas aeruginosa*	[59]
*Honeydew honey*: Antibacterial activity was detected in honeydew honey against Gram-positive *Staphylococcus aureus* and Gram-negative *Pseudomonas aeruginosa*	[60]
*Manuka*: Protection of human dermal fibroblasts shown against oxidative damage by activation of AMPK/Nrf2 signalling improving antioxidant response and mitochondrial function, and by protection against oxidative damage and improving the process of skin wound healing	[61]

### 4.1. Antifungal activity of honey

Hau-Yama et al assessed the antifungal potential of honey made by the stingless bee species *Melipona beecheii* against the opportunistic yeast species *Candida albicans* [51]. It was noticed that honey concentrations at 20% (v/v) and higher were able to inhibit the growth of *C. albicans*, and could negatively alter the integrity and morphology of the pathogen's cell wall structure. The honey at a concentration of 35% (v/v) demonstrated cidal properties within *in vitro* tests [51].

Fernandes et al assessed the antimycotic potential of Portuguese honeys on *Candida spp*. in both planktonic and biofilm assessments, as well as the *S. aureus* and *P. aeruginosa* strainsas compared to results generated from Manuka honey [52]. Researchers observed that honey from the Portuguese Heather (*Erica cinereal*) was similar to Manuka honey in its physiochemical properties; specifically, it had almost identical phenolic and flavonoid profiles. All clinical isolates/reference strains of *C. albicans* demonstrated a potent susceptibility to all five samples of Portuguese Heather honey. The concentration in the range of 50–75% w/v was found to be the most efficient in reducing biofilms caused by *Candida spp*., especially by *C. tropicalis* [52].

Hamid et al performed an *in vitro* evaluation of the susceptibility and sensitivity of selected pathogenic microbes responsible for otomycosis, such as the fungal species *Aspergillus niger* and the yeast *Candida albicans*, to three varieties of local Malaysian honey–Taulang, Acacia, and Kelutut [53]. In the antimicrobial assays, the results concluded that all three types of honey demonstrated *in vitro* antimycotic properties, with distinct potentials to further develop novel antifungal treatments for conditions such as otomycosis [53].

Though *Candida spp*. is one of the most common fungal infections seen within humans, there does appear to be some bias towards fungal species that are subject to the assessments of different types of honey. Despite this discrepancy in the literature, studies do exist that explored the antifungal potential of honey on species of fungi and not solely *Candida spp*. Vică et al assessed the antimycotic properties of Romanian honey from North-West and Central Romania and the Alba County on six species of fungal pathogen: *Aspergillus niger*, *Aspergillus flavus, Candida albicans, Penicillium chrysogenum, Rhizopus stolonifer*, and *Fusarium oxysporum* [54]. All honey samples–12 North-West and Central, and 13 Alba County – demonstrated distinct antimycotic properties, though the potency was noted to vary between the geographical locations of origin. Vica et al concluded that the more sensitive species were *Penicillium chrysogenum, Rhizopus stolonifer*, and *Fusarium oxysporum*. Additionally, the report indicated that there was a significant connection between the geographical origin of the honey sample, the fungal strain, and the antimitotic property – showing that these factors and the origin and pathogen strains did actively influence the inhibition diameters [54].

Azonwade et al conducted an analysis and assessment of the antifungal properties of sixty kinds of honey samples harvested from 30 distinct phytogeographical locations in Benin–Sudanian, Sudano-Guinean, and Guinean–during the dry and wet seasons on four reference strains of *Aspergillus* including *niger, parasiticus, fumigatus*, and *clavatus* [55]. The experimental outcome concluded that though the antioxidant profiles restricted the honey samples to a dose dependency, all samples demonstrated clear antimycotic properties on all *Aspergillus* spp., with no particular distinctly sensitive strain. The researchers observed that the honey samples collected during the wet climatic conditions significantly demonstrated more potent antifungal properties than those collected during the dry season [55]. As previously stated, though literature does exist that explored the effects of various honey samples on fungal pathogens, not solely *Candida* sp., the reports are still limited, with the area of study seemingly underappreciated within a clinical research context for novel antimycotic agents.

## 5. Infection Control related to Healing of wounds infected by antibiotic-resistant pathogens

Marimuthu et al analyzed Malaysian Stingless-Bee Kelutut honey and compared it to medicinal-grade Manuka honey to understand its potential to act as a treatment agent for wounds, specifically for the healing of diabetic foot ulcer wounds due to increasing complications in the treatment of such wounds as a result of an increasing resistance to antibiotics. In a randomised study, 60 adult patients were chosen, each with chronic diabetic foot ulcers, into two groups, where each group was applied with either Kelutut or Manuka honey-based dressings for 14 days after maggot therapy debridement [56]. The results of this study showed that the group of patients treated with the Kelutut honey had an average total wound size reduction of 42%, compared to the 7.5% reduction in wound size observed within the medicinal-grade Manuka honey group. Moreover, the Manuka honey group wounds were observed to have a greater exudate rate, or leaking of fluid from the injury, than in wounds of the Kelutut honey group [56]. The report concluded that patients whose wounds were dressed with manuka honey experienced a mildly painful sensation, at an average pain score of 5, lasting approximately 30 minutes, whereas the group that was treated with Kelutut honey experienced no such sensation at all.

In another research project, Skadiņš et al evaluated the potential of Latvian mono-floral honey as an agent to aid chronic wound healing within patients by analysing the honey's antimicrobial and antibiofilm properties [57]. Latvian mono-floral honey samples were harvested and evaluated alongside 800+ MGO manuka honey against clinical strains and isolates of *Escherichia coli*, extended spectrum beta-lactamase producing *Escherichia coli*, methicillin-resistant *Staphylococcus aureus, Pseudomonas aeruginosa, Staphylococcus aureus*, and *Candida albicans*. Prolific antibacterial properties were observed in 11 of the 40 varieties of honey from Latvia across all the tested strains of bacteria; additionally, several of the honey varieties demonstrated a greater effect on all test strains compared to the manuka honey samples. There was no comment on the antifungal potential of the Latvian honey due to inconclusive results [57].

Consistently, Yamani and Fakiah evaluated the acute excisional wound healing capacity of a formulation of Tahl honey, whey protein, and collagen in male rat models compared with treatment using Manuka honey and a Povidone 5% iodine ointment [58]. Twenty-four male rats were divided into four groups (n = 6) as follows: group 1 (no treatment/negative control), group 2 (Manuka honey), group 3 (Povidone 5% iodine ointment), and group 4 (Formulation of Tahl honey, whey protein, and collagen). All rats received 1cm diameter circular wounds into their dorsal skin, all wounds were washed with a saline solution, and the respective test material was applied to them and covered. All rats were individually caged and Groups 2, 3, and 4 had their materials reapplied every 2 days post-operation. The experimental honey formulation indicated a safe therapeutic potential with an accelerated wound contraction and closure rate, while no indication of infection due to bacterial or fungal pathogens was observed across the 18 days post-operation comparable to an antibiotic material [58].

## 6. Mechanism of Honey's Therapeutic properties

**Figure 2.**
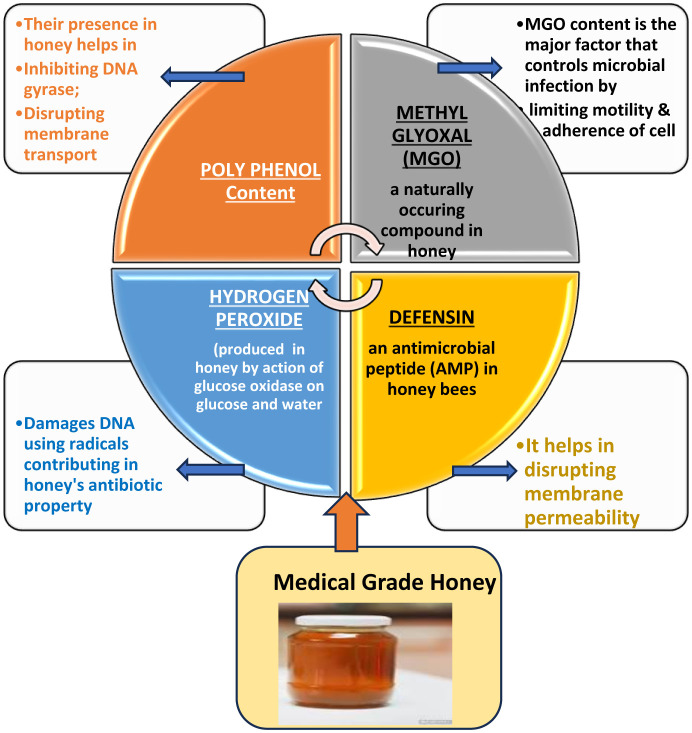
Mechanism of infection control by bioactive compounds of honey.

The exact mechanism by which these honeys conduct and coordinate infection control and wound healing is not precisely known, or the subject is of limited analysis, such as phytochemical and phenolic compound fingerprinting. However, a proposed mechanism (Figure 2) of action by which varieties of medical grade honey execute their bioactive properties, specifically antibacterial, antioxidative, and wound healing properties, is dependent on the hydrogen peroxide (H_2_O_2_) concentrations in the material, which is also aided by the varying and diverse polyphenol content [59],[60].

As Manuka honey is a mono-floral variety of honey that has flagged itself within clinical studies, the composition of the material is known, including its phytochemical makeup, and the mechanisms by which it conducts wound healing are more comprehensively understood. Alvarez-Suarez et al evaluated the capacity of Manuka honey's antioxidative nature to protect against oxidative-related damage, as well as its capacity to improve wound healing [61].

Through their evaluation, researchers concluded with several observations: Manuka honey ceased cell progression towards apoptosis; protected cells from intracellular reactive oxygen species generation; controlled damage to cells from oxidative damage associated with lipids and proteins; a potential means by which manuka honey can improve wound healing; the activation of 5′ AMP-activated protein kinase (AMPK) phosphorylation; overexpression of the nuclear factor erythroid 2-related factor (Nrf2) signalling pathway; and an improved activation of the superoxide dismutase (SOD) and catalase (CAT) antioxidative enzymes were reported [61]. Moreover, a note was made of its protective effect over mitochondrial protection, which could be the result of the intrinsic Manuka honey polyphenols scavenging free radicals associated with the mitochondrial pathways and the regulating effect of polyphenols on mitochondrial pathways. The membrane potential control, electron transport, and apoptosis create a preservative effect on the organelle, thus encouraging and promoting total cell proliferation and eventual wound closure [61],[62].

A study by Sell et al was analogous to a report published by Alvarez-Suarez et al. The potential of Manuka honey and platelet-rich plasma was evaluated in wound healing. They studied the potential responses of human fibroblasts, macrophages, and endothelial cells towards platelet-rich plasma, specifically plasma rich in growth factors, and Manuka honey. All three cell types positively responded to the presence of the plasma rich in growth factor medium; however, they observed a stronger positive response to the combination of plasma rich in growth factors and manuka honey. The highest response came from the human fibroblasts, which demonstrated an increase in proliferation, increased collagen matrix production, as well as increased migration into an *in vivo* wound model that was supplemented with plasma rich with growth factors and Manuka honey [63]. Further research is needed to definitively rule out if there is any negative impact of the application of topical formulations of an antimicrobial nature, as their use is still debated within standard and alternative wound care and the data is still inconclusive [64].

## 7. Honey used in pharmaceutical products

As discussed in the above sections, research studies have testified and clinically analyzed the many exploitable properties of honey on different types of infections and cell types either *in vitro* or *in vivo*. However, all these clinical evaluations share one similar fact throughout: researchers have subjected media, models, cells, and microorganisms to either a pure sample of honey or a diluted solution made simply by mixing deionised water. A study conducted by Hossain et al set out to evaluate prototype topical formulations made using four varieties of western Australian honey – two manuka, a Jarrah, and a Coastal Peppermint variety of honey; New Zealand manuka honey was included in the samples as a comparable control. The aim was to assess the bioactive antibacterial and antioxidative properties in diluted samples against their corresponding pure honey material and assess if their formulations retained a comparable bioactivity similar to the pure samples [65].

Researchers in their investigation subjected the pure honey samples and their formulations to the Ferric Reducing Antioxidant Power (FRAP) and High-Performance Thin-Layer Chromatography coupled with 2,2-Diphenyl-1-Picrylhydrazyl (HPTLC-DPPH) to determine the antioxidative power activity and identification of phenolic compounds [66]–[68], while the same samples were subjected to an Agar-Overlay Assay to determine the antimicrobial power against a few bacterial species, namely *Staphylococcus aureus, S. epidermidis, Enterococcus faecalis, Micrococcus luteus, Escherichia coli, Pseudomonas aeruginosa, Acinetobacter baumannii*, and *Klebsiella pneumoniae*. The evaluation report of all honey-loaded formulations of each type of honey demonstrated comparable bioactivities, both antibacterial and antioxidative, compared with their corresponding pure material types [65].

Similar to the work of Hossain et al, Manuka honey has been shown to synergistically work alongside other known and standardised materials and agents of chemotherapeutic interventions–especially within the veterinary context. A study produced by Váczi et al investigated the efficacy and power of Manuka honey obtained as a commercial brand Activon that contained 25 g of pure medicinal quality Manuka on 14 isolates of the fungal pathogen *Malassezia pachydermatis*, which is a secondary causative agent of fungal infections after yeast infections within cats and dogs, alongside the prescription of four standard azole antifungal agents (clotrimazole, fluconazole, itraconazole, and miconazole) [69]. Honey was diluted in test tubes containing Sabouraud dextrose broth containing Tweens 40 and 80 to concentrations from 800–12.5 mg/mL. Researchers used a modified CLSI M27–A3 method for antifungal susceptibility, and a checkerboard method was used to separately determine the antifungal effect of the materials, the azole antifungals, and the manuka honey; more importantly, in this investigation, the antifungal power and effect of the materials were investigated in combination. The assessment of the data demonstrated an enhanced, or additive, effect that was imposed upon the azole antifungals by the manuka honey itself, and that a mutual combination of the materials together could produce a greater antifungal property compared to their individual activity.

Although the published reports are limited on the applications of honey on fungal pathogens, or its ability to produce antifungal properties, the research by Váczi et al demonstrated the synergistic property of Manuka honey with only azole antifungal chemical compounds. A similar demonstration of the synergistic properties of Manuka honey was shown in a research published by Harrison et al [70]; however, this synergy explored the antibacterial and antibiofilm activity of acetic acid and vinegar combined with different types of medical-grade honey.

It should be noted that, especially with other types of honey beyond manuka honey, the appeal of honey that is used in new pharmaceutical products is associated with their origin of varied floral species. A variety of floral species increases the constituent concentration of organoleptic and bioactive compounds in honey. Therefore the socio-demographic profiles inform consumers and manufacturers of the apparent health benefits corresponding to that of honey [71]. As previously stated, the bioactivity of polyphenols with certain honey types of multi-floral origins and the concentration of H_2_O_2_ are higher compared to mono-floral types of honey such as Manuka honey [72]. A brief overview and list of current pharmaceutics that contain honey as a key ingredient is presented in Table 3.

**Table 3.** List of some of the commercial pharmaceutical products with honey as a key healing constituent in their formulations.

Honey-Based Pharmaceutical Product	Application and Benefits	Active components used in Formulation	Product-Manufacturer/supplier	Reference
MediHoney Barrier Cream	Reduces inflammation; prevents maceration, excoriation, and irritation, particularly as the result of incontinence and diarrhoea; moisturises and protects dry skin	Manuka from *Leptospermum scoparium* (100%)	Derma Sciences	[73]
Manuka IG Max wounds dressing	Facilitates wound healing; hydrates wound; promotes autolytic debridement; facilitates exudate transfer to secondary dressing	Manuka impregnated customised gauze	IMS Euro Ltd	[74]
Actilite	Antibacterial; promotes cell proliferation/new cell growth; reduces scar formation; controls wound moisture levels; prevents maceration of wound	Viscose net dressing, coated with Manuka honey (99%) + Manuka oil (1%).	Advancis Medical	[75]
MelMax® dressing	Antibacterial; regulates enzymatic balance within wound; antioxidative and prevents oxidative damage to the wound	Dressing impregnated with a metal-ion formulation + medical grade buckwheat honey (source of phenolic compounds for superior anti-oxidant activity)	Principelle	[76]
HoneySoft	Antibacterial; protects and hydrates newly closed wounds; repairs irritated skin; promotes microbial balance of the skin	Chilean multifloral honey, acetate dressing impregnated with honey	Taureon	[77]
Nature's Gold TGA Listed Skin Cream with Manuka Honey	Treats rashes, eczema, dermatitis, psoriasis acne, dry skin, cracked heels, bruises, small cuts and abrasions.	Certified premium Manuka honey (10%) skin cream, a blend of Australian bush botanicals including macadamia nut oil, rosehip oil, safflower oil and avocado oil	Nature's Gold	[78]
L-Mesitran® Soft	Promotes wound healing; promotes cell migration; antibacterial; aids wound bed preparation; facilitates and promotes epithelisation; promotes moist wound healing environment	Medical Grade Honey (40%), Lanolin, antioxidants vitamins E + C, glycol	H&R Healthcare;	[79]
Activon Tube	Recommended for debriding necrotic tissue, or for topping up dressings where honey has been removed with exudate	Medical-grade Manuka honey (100%)	Advancis medical uk	[80]
Comvita Medihoney Antibacterial Wound Gel	wound gel containing sterilised medical grade Manuka honey; promotes wound healing and reduces the risk of infection for broken eczema prone skin and skin splits.	Mel (Medihoney® Antibacterial Honey), and natural gelling agents.	Comvita	[81]
Algivon Plus	Wound Dressing	Alginate dressing impregnated with 100% Manuka Honey	Advancis medical uk	[82]
REVAMIL® Hydrophilic Wound Gel	Revamil's enzyme-rich honey provides antibacterial protection. The low pH of the honey creates an acidic environment so that the wound heals faster	Medical Grade Honey (100%) with a high content of the honey enzyme glucose oxidase	BFactory Health Products B.V.	[83]
Melladerm® Plus,	Bacteriostatic wound gel for the treatment of superficial (contaminated) chronic, oncologic and/or acute wounds.	Honey 45%, from a multi flower mountain region known as a free pesticide and pollution area + glycerine, glycol	SanoMed Manufacturing bv,	[84]
L-Mesitran Ointment	Ointment is suitable for the treatment of chronic, oncological and acute wounds like abrasions, cuts and burns.	Medical Grade Honey (48%), lanolin, *Aloe barbadensis*, *Calendula officinalis*, sunflower oil, cod liver oil, Vitamin C + E, Zinc Oxide	Mesitran	[85]

It is interesting to note that different formulations were prepared for specific applications and used different percentages of honey. Honey based products designed especially for wound care [86],[87] should consider an important point, namely that a high concentration of honey (>50%) is associated with experiencing more pain during the application of pure honey, which is attributed to a stronger osmotic activity compared with lower concentrations.

## 8. Conclusions and prospects

Several studies for the screenings of many types honey have been performed in the last few years to recognize new sources of medical-grade honey. This is useful in the search for new antibiotics. In most studies, the antibacterial activity of locally sourced varieties of honey was usually examined using a benchmark of different levels of MGO, which is similar to the categorization of Manuka honey as a unique manuka factor (UMF) [30],[32],[33],[59]. Clinically, honey is most often used in its natural form in wound care, and it can be diluted for applications on different wound types depending on the severity of the infection and the amount of inflammation in exudating wounds. However, it is important to note that the concentration of honey should be used to a certain level, which gives the maximal rate of H_2_O_2_ production for effective antibacterial activity in honey [39],[41].

Honey is a natural nutraceutical and can be used in combinational therapeutic approaches [88]. Hydrogen peroxide has been reported as a potential wound therapeutic target [89], in which the stabilization and controlled release of H_2_O_2_ for wound treatment applications could be achieved through the use of micro-encapsulated hydrogen peroxide [90]. As mentioned in the introduction section, the need for new antibiotics to strengthen the IPC capacity in healthcare facilities could help to set a strategy to reduce the spread of antimicrobial resistance and infections associated with healthcare. Non-manuka conventional varieties of honey should also be explored for specific treatments, similar to the management cases of side/after effects of radiotherapy and radio with chemotherapy-induced oral mucositis. A systematic review analysed 17 randomized trials on participants who had received the treatment of neck and head cancer either by radiotherapy or combined radiotherapy with chemotherapy. Regarding the clinical efficacy, there were differences observed. In the study, manuka honey was not effective to treat mucositis (n = 4 studies), while trials using conventional honey were effective (n = 13 studies). Thus, the type of honey may explain the divergent results of trials in this area [91].

After reviewing the results reported in recent investigations on different floral varieties of honey sourced from various geographical regions, including Australia, New Zealand and Mediterranean countries, there is certainly a potential for honey as a complimentary antibiotic. In particular, some reports are very significant where honey was obtained from the nectors of local medicinal plants. In the current occurrence of antibiotic resistance for prescribed standard antibiotics, the availability of therapeutic quality honey, regardless of its floral variety or geographical location, provides new resources for the development of natural and non-chemically synthesized antibacterial and antifungal options. Currently, several types of manuka honey labelled with the source of their production are available to buy in health shops and supermarkets; additionally, various manuka brands are sold online globally. There could be authenticity issues with honey (adulteration and dilution with sugar solutions), especially the relatively expensive Manuka honey. For example, if there are tons of Manuka honey products with a specific brand name sold globally, as compared to its actual lesser production each year, there could be a problem with the product being sold. The prospects for the beneficial uses of a natural resource require further investigation and analyses to aid in the understanding of the therapeutic applications of honey sourced from different floral varieties, particularly for the treatment of chronic and non-healing wounds.
